# Risk factors and survival outcomes of metachronous contralateral upper tract urothelial carcinoma

**DOI:** 10.1038/s41598-020-73699-5

**Published:** 2020-10-06

**Authors:** Kan Wu, Jiayu Liang, Yiping Lu

**Affiliations:** Department of Urology, Institute of Urology, West China Hospital, Sichuan University, 37 Guoxue Lane, Chengdu, 610041 Sichuan China

**Keywords:** Urological cancer, Outcomes research

## Abstract

Because population-based risk estimates for metachronous contralateral UTUC are lacking. In this study, we aimed to evaluate the risk and survival of metachronous contralateral upper tract urothelial carcinoma (UTUC) on a large population-based level. A total of 23,075 patients were identified from the Surveillance, Epidemiology, and End Results database (1973–2015), 144 (0.6%) patients developed metachronous contralateral UTUC (median of 32 months after diagnosis). The cumulative incidence at 10, 20, and 30 years of follow-up was 1.1%, 1.6%, and 2.6%, respectively. We applied Fine and Gray’s competing risk regression model to determine the risk factors of a new contralateral, metachronous UTUC. The competing risk regression model demonstrated that older age (hazard ratio [HR] 0.75; 95% CI 0.67–0.85) and larger tumor size (HR 0.61; 95% CI 0.39–0.97) were associated with a significantly decreased risk of metachronous contralateral UTUC. However, bladder cancer presence was an independent risk factor for the development of contralateral tumors (HR 2.42; 95% CI 1.73–3.37). In addition, we demonstrated developing contralateral UTUC was not associated with poor prognosis by using Kaplan–Meier and multivariable analysis. Our findings suggest that metachronous contralateral UTUC is comparatively rare, and has not impact on survival. Importantly, patients with younger age, small tumours, and the presence of bladder cancer were more likely to develop a contralateral tumor, which may provide a rationale for lifelong surveillance in high-risk patients.

## Introduction

Upper tract urothelial carcinoma (UTUC) is a relatively rare neoplasm, representing about 5–10% of all urothelial cancers^1^. The gold-standard treatment of UTUC is radical nephroureterectomy (RNU) with excision of the bladder cuff^2^. The development of metachronous contralateral UTUC after RNU is extremely rare, with an incidence rate of 0.8–6.9%^3–9^. However, the risk of consequent compromised renal function can be very grave, resulting in inevitable permanent dialysis. Thus, the risk stratification for predicting subsequent contralateral UTUC is necessary for planning the routine follow-up surveillance and the proper treatment.

Only a few recent studies have focused on the characteristics and risk factors of metachronous contralateral UTUC^8–13^, These available studies are limited to small sample sizes or relatively short follow-up. Furthermore, due to the rarity of contralateral cancer, information on the survival outcome of metachronous contralateral UTUC is lacking. Therefore, in a large, population-based cohort, we sought to identify the risk factors for developing metachronous contralateral UTUC and attempt to describe survival outcomes among patients who diagnosed with unilateral UTUC and contralateral tumors.

## Methods

### Patient population

We identified patients diagnosed with UTUC from the National Cancer Institute’s Surveillance, Epidemiology, and End Results (SEER) database between January 1973 and December 2015. Metachronous contralateral UTUC is defined as urothelial carcinoma diagnosed in the contralateral upper urinary tract after the first diagnosis of unilateral UTUC.

Inclusion criteria included the following: patients with a primary site labeled as “C65.9 Renal pelvis, or C66.9 Ureter”, and the histology codes of transitional cell carcinoma including: 8120/2, 8120/3, 8122/3, 8130/2, 8130/3, and 8131/3 (Detailed definition of ICD-O-3 SEER Site/Histology Validation List [https://seer.cancer.gov/icd-o-3/]). Only microscopically confirmed cases of UTUC were included. Patients < 18 years or with unknown information regarding laterality and race were excluded.

From the SEER database, 26,117 patients diagnosed with UTUC were eligible for inclusion. Subsequently, to assess the risk of developing metachronous contralateral UTUC, we excluded patients presenting with: follow-up < 3 months, unknown survival months, or synchronous bilateral UTUC.

### Statistical analysis

The Kaplan–Meier method was used to calculate the cumulative incidence of metachronous contralateral UTUC. Standardized incidence ratio (SIR) was defined as the ratio of observed cases to expected cases in the general population. Chi-square and *t *tests were used to assess the differences in baseline clinicopathologic characteristics between patient groups. We used univariate and multivariate Fine and Gray’s competing risk regression models to identify risk factors associated with a new contralateral, metachronous UTUC. Survival curves were performed by using the Kaplan–Meier method, and the differences between the curves were compared by using the log-rank test.

P values < 0.05 were considered statistically significant and P-values were two-sided. Statistical Analyses were performed with SPSS software version 23.0 (IBM, Armonk, NY, USA), apart from the competing risks analysis, which was performed by R software (version 3.5.1; R Foundation) using the cmprsk package.

### Statement of ethics

We signed the SEER Research Data Agreement (No. 12587-Nov2019) and further searched for data according to the approved guidelines. The SEER data were open available and patients’ records are anonymous, therefore, this study was deemed exempt from review by our institutional review board.

## Results

### Study population

A total of 23,075 patients were diagnosed with unilateral UTUC and a follow-up period more than 3 months, among them, 144 (0.6%) patients subsequently developed a new metachronous contralateral UTUC (Table 1). The median age of our entire cohort was 72 years (interquartile range [IQR] 64–79 years). In all 14,002 (60.7%) patients were men and 9073 (39.3%) were women. White, and other race accounted for 89.2%, and 10.8% of the population, respectively.

**Table 1:** Clinicopathologic characteristics of patients who developed a new, metachronous contralateral UTUC.

Characteristic	Overall (N = 23,075)	Metachronous, contralateral UTUC		*P*
		No (n = 22,931)	Yes (n = 144)	
Age, year, median	72	72	68	< 0.001
**Sex**				
Men	14,002 (60.7)	13,909 (60.7)	93 (64.6)	0.336
Women	9073 (39.3)	9022 (39.3)	51 (35.4)	
**Race**				
White	20,583 (89.2)	20,458 (89.2)	125 (86.8)	0.353
Other	2492 (10.8)	2473 (10.8)	19 (13.2)	
**Bladder cancer presence**				
No	16,703 (72.4)	16,635 (72.5)	68 (47.2)	< 0.001
Yes	6372 (27.6)	6296 (27.5)	76 (52.8)	
**Laterality**				
Left	11,627 (50.4)	11,559 (50.4)	68 (47.2)	0.446
Right	11,448 (49.6)	11,372 (49.6)	76 (52.8)	
**Location**				
Pelvis	14,244 (61.7)	14,164 (61.8)	80 (55.6)	0.126
Ureter	8831 (38.3)	8767 (38.2)	64 (44.4)	
**Extent of disease**				
Local	8314 (36.0)	8242 (35.9)	72 (50.0)	< 0.001
Regional	12,039 (52.2)	11,977 (52.2)	62 (43.1)	
Distant	1756 (7.6)	1756 (7.7)	0 (0)	
Unknown	966 (4.2)	956 (4.2)	10 (6.9)	
**Surgery**				
Nephroureterectomy	12,382 (53.7)	12,319 (53.7)	63 (43.8)	0.003
Nephron-sparing surgery	1964 (8.5)	1947 (8.5)	17 (11.8)	
Tumor local excision	3468 (15.0)	3433 (15.0)	35 (24.3)	
No	2064 (8.9)	2057 (9.0)	7 (4.9)	
Unknown	3197 (13.9)	3175 (13.8)	22 (15.3)	
**Pathology grade**				
I–II	6823 (29.6)	6760 (29.5)	63 (43.8)	0.001
III–IV	13,275 (57.5)	13,208 (57.6)	67 (46.5)	
Unknown	2977 (12.9)	2963 (12.9)	14 (9.7)	
**Tumor size, cm, median**	3.5	3.5	2.8	< 0.001
≤ 3 cm	7028 (30.5)	6976 (30.4)	52 (36.1)	0.002
> 3 cm	8582 (37.2)	8549 (37.3)	33 (22.9)	
Unknown	7465 (32.3)	7406 (12.9)	59 (41.0)	

Data are presented as n (%) unless noted otherwise.

*UTUC* upper tract urothelial carcinomas.

Among the 144 patients with metachronous contralateral UTUC, 76 (52.8%) patients had a history of bladder cancer, compared with 27.5% of patients with unilateral UTUC (6296 of 22,931) (*P* < 0.001). In addition, metachronous contralateral tumors were more common among patients with younger age (68 vs 72 years; *P* < 0.001), who had localized disease (*P* < 0.001), lower pathology grade (*P* = 0.001) or smaller tumor size (2.8 vs 3.5 cm, *P* < 0.001). Notably, 24.3% of patients with contralateral tumors had received tumor local excision (such as polypectomy thermal ablation, and laser ablation), compared with 15.0% of patients with unilateral UTUC (3433 of 22 931) (*P* = 0.003).

### Occurrence pattern and risk factors of metachronous contralateral UTUC

**Figure. 1:**
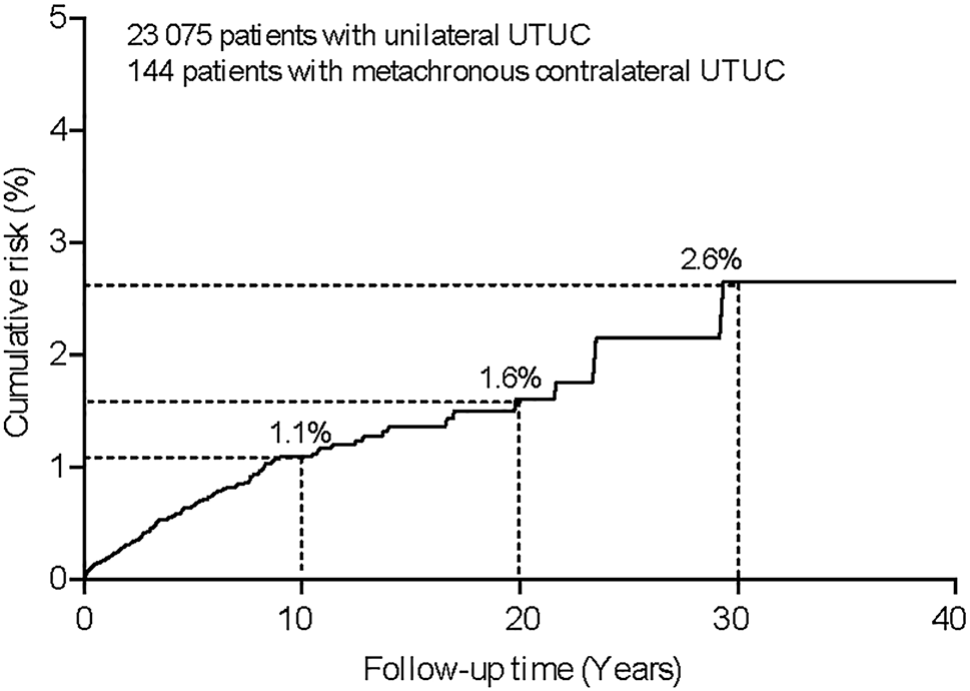
Cumulative incidence of metachronous contralateral upper tract urothelial carcinomas (UTUC).

After a median interval of 32 months (IQR 9–71 months), 144 (0.6%) patients subsequently developed a new metachronous contralateral UTUC. A total of 101 (70.1%) new metachronous contralateral tumors were detected within 5 years, 28 (19.4%) between 5 and 10 years, and 15 (10.4%) after 10 years of primary diagnosis. The 10-, 20-, and 30-year cumulative incidence rates for metachronous, contralateral UTUC were 1.1%, 1.6%, and 2.6%, respectively (Fig. 1). We treated death as a competing event, and used the competing risks regression model to identify risk factors of metachronous contralateral UTUC (Table 2). In the univariate analysis, age at initial diagnosis (*P* < 0.001), history of bladder cancer (*P* < 0.001), tumor stage (*P* = 0.008), the mode of surgery (*P* < 0.05), tumor grade (*P* = 0.004) and tumor size (*P* = 0.004) were associated with the development of contralateral disease for patients with UTUC. However, in the multivariable analysis, older age at initial diagnosis (hazard ratio [HR] per 10-year age increase, 0.75; 95% confidence interval [CI] 0.67–0.85), and larger tumor size (> 3 cm vs. ≤ 3 cm, HR 0.61; 95% CI 0.39–0.97) were associated with significantly decreased risk of metachronous contralateral tumor. In addition, the presence of bladder cancer was an independent risk factor of the occurrence of contralateral UTUC in the multivariable model (HR 2.42; 95% CI 1.73–3.37). However, sex, race, laterality, tumor location, stage, the mode of surgery and pathology grade were not associated with the development of contralateral disease.

**Table 2:** Univariate and multivariate analysis for the development of new, metachronous contralateral UTUC.

Variable	Univariate		Multivariate	
	HR (95% CI)	*P*	HR (95% CI)	*P*
Age	0.76 (0.67–0.86)	< 0.001	0.75 (0.67–0.85)	< 0.001
**Sex**				
Men	Ref			
Women	0.85 (0.61–1.20)	0.360		
**Race**				
White	Ref			
Other	1.28 (0.79–2.08)	0.310		
**Bladder cancer presence**				
No	Ref		Ref	
Yes	2.74 (1.98–3.80)	< 0.001	2.42 (1.73–3.37)	< 0.001
**Laterality**				
Left	Ref			
Right	1.13 (0.82–1.57)	0.450		
**Location**				
Pelvis	Ref			
Ureter	1.29 (0.93–1.79)	0.130		
**Extent of disease**				
Local	Ref		Ref	
Regional	0.63 (0.45–0.89)	0.008	0.76 (0.53–1.10)	0.150
Distant^a^	NA	NA	NA	NA
Unknown	1.23 (0.64–2.39)	0.530	1.70 (0.81–3.53)	0.160
**Surgery**				
Nephroureterectomy	Ref		Ref	
Nephron-sparing surgery	1.68 (0.99–2.88)	0.056	1.41 (0.81–2.47)	0.220
Tumor local excision	1.73 (1.14–2.62)	0.001	1.27 (0.81–1.98)	0.300
No	0.65 (0.30–1.41)	0.270	0.63 (0.28–1.45)	0.280
Unknown	1.16 (0.71–1.88)	0.550	0.87 (0.47–1.62)	0.670
**Pathology grade**				
I–II	Ref		Ref	
III–IV	0.60 (0.43–0.85)	0.004	0.86 (0.60–1.23)	0.410
Unknown	0.55 (0.31–0.98)	0.043	0.74 (0.41–1.33)	0.310
**Tumor size**				
≤ 3 cm	Ref		Ref	
> 3 cm	0.53 (0.34–0.81)	0.004	0.61 (0.39–0.97)	0.037
Unknown	0.99 (0.68–1.44)	0.960	1.10 (0.69–1.75)	0.690

Variables with p < 0.05 in univariate analysis were included in the multivariate model.

*UTUC* upper tract urothelial carcinomas, *CI* confidence interval, *HR* hazard ratio.

^a^We excluded patients with distant metastasis in this analysis, due to patients with metachronous contralateral UTUC did not have distant metastatic disease.

Furthermore, we assessed the risk of new, metachronous contralateral tumors in unilateral UTUC patients compared to the risk of UTUC in the general population. The overall risk of secondary UTUC in unilateral UTUC patients was more than 18-fold greater than the risk of UTUC in the general population (SIR 18.5; 95% CI 15.5–21.5). For these patients, the risk of contralateral disease remained significantly elevated even after long-term follow-up (30 years), although the magnitude of the SIR elevation decreased obviously over time [0–4 years after the first diagnosis: SIR (95% CI), 50.5 (40.2–60.8); 5–9 years after the first diagnosis: SIR (95% CI), 14.9 (10.5–20.6); ≥ 10 years after the first diagnosis: SIR (95% CI), 4.2 (2.4–7.0)].

### Follow-up outcome

Among the 144 patients who developed a new, metachronous contralateral UTUC, 52.8% were the same tumor stage, 15.3% were of a higher stage, 17.4% were of a lower stage, and 14.6% were unstaged. Of these 107 patients who had metachronous tumor and pathology grade data for both tumors, 49.5% were the same tumor grade, 29.9% were of a higher grade, and 20.6% of these patients had a lower grade. For patients who developed a metachronous contralateral UTUC and initially treated with RNU (63 patients), 33.3% (21/63) underwent a second RNU, 9.5% (6/63) received partial nephrectomy or ureterectomy, 34.9% (22/63) underwent tumor local excision, and 19% (12/63) did not have further surgical treatment.

**Figure. 2:**
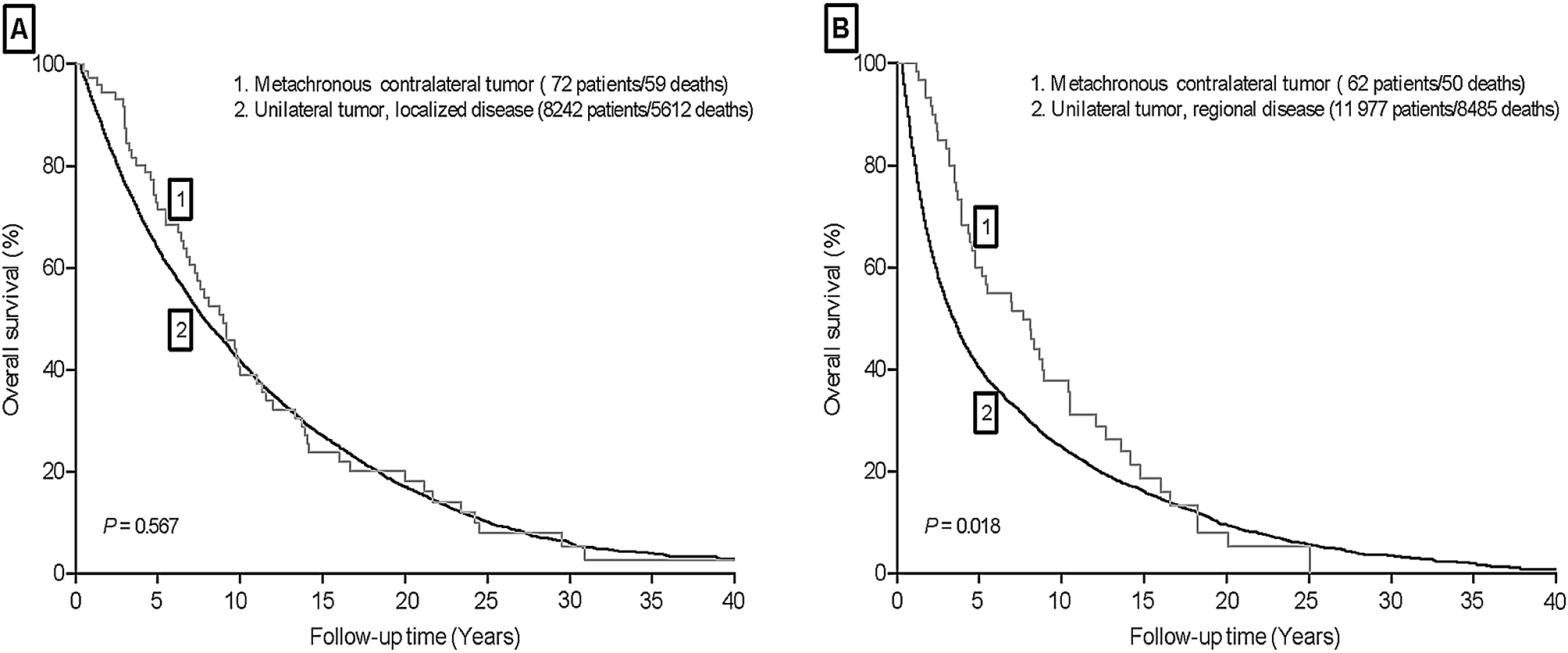
Overall survival of patients who initially presented with unilateral or metachronous contralateral upper tract urothelial carcinomas (UTUC), stratified by extent of the disease at diagnosis.

Among the patients who developed a new, metachronous contralateral UTUC and patients with unilateral UTUC, 116 (80.6%) and 16 398 (71.5%) patients died during follow-up, respectively. Survival analysis based on stage was performed on patients with contralateral tumors and patients with unilateral UTUC (Fig. 2). Among patients who had localized disease, the estimated overall survival at 5 years was 71.4% for metachronous contralateral tumor versus 63.6% for patients with unilateral UTUC (*P* = 0.567, Fig. 2A). Interestingly, patients with contralateral UTUC and who had regional stage had better survival compared with unilateral UTUC patients with the regional disease (5-year overall survival rate: 60.0% vs 40.2%; *P* = 0.018, Fig. 2B). However, in multivariable Cox regression analysis for overall survival in patients with unilateral UTUC at initial diagnosis (Table 3). The development of new, metachronous contralateral tumor was not associated with survival outcomes of patients with UTUC regardless of tumor stage (localized stage: HR 1.28; 95% CI 0.99–1.66; regional stage: HR 0.80; 95% CI 0.61–1.06; unknown stage: HR 0.79; 95% CI 0.37–1.69).

**Table 3:** Overall survival according to multivariate analysis in different groups.

	Metachronous, contralateral UTUC	5-year overall survival rate, %	*P* value	HR (95% CI)
Localized stage	Yes	71.4	0.060	1.28 (0.99–1.66)
	No	63.6		
Regional stage	Yes	60.0	0.122	0.80 (0.61–1.06)
	No	40.2		
Unknown	Yes	–	0.543	0.79 (0.37–1.69)
	No	42.9		

HR in multivariate analysis were adjusted for age, sex, race, bladder cancer presence, laterality, tumor location, the mode of surgery, tumor grade, tumor size.

*UTUC* upper tract urothelial carcinomas, *HR* hazard ratio, *CI* confidence interval.

## Discussion

In this large population-based cohort of more than 20,000 patients initially diagnosed with unilateral UTUC, we find that unilateral UTUC patients have an 18.5-fold excess risk of developing a new metachronous contralateral tumor compared to the risk of UTUC in the general population. This excess risk was highest within the first 5 years after an initial diagnosis of UTUC and decreased obviously thereafter. The 30-year cumulative incidence for developing a new metachronous contralateral UTUC was 2.6%. Other new findings from this study include that older age at initial diagnosis, and larger tumor size are associated with a decreased risk of metachronous contralateral UTUC. However, we find that bladder cancer presence is a very important risk factor for contralateral UTUC development. In addition, this study indicates that the development of a new metachronous contralateral UTUC is not associated with poor survival compared with patients with unilateral UTUC.

Our findings on crude incidence rates (0.6%) of metachronous contralateral UTUC in this cohort are lower than the incidence reported by most Chinese studies with comparable information^8,9,12^. The 30-year cumulative incidence risk (2.6%) we report is, however, in line with the estimates of metachronous contralateral tumors that have been reported by Swedish study during a 28-year period^10^. There are several possible interpretations for the discrepancy with the other Chinese studies. First, Chinese analyses of subsequent contralateral UTUC after an initial diagnosis often include many patients with renal insufficiency commonly caused by the consumption of Chinese herbs containing aristolochic acid which was an independent risk factor of metachronous contralateral UTUC^8,9,12,14,15^, but aristolochic acid-induced UTUC was not common in the American population. Second, some prior studies were limited by small sample sizes or the median follow-up < 5 years, which might overestimate disease occurrence^9,11–13^. Because we found that the increased risk of contralateral tumor was highest within the first 5 years after an initial diagnosis of UTUC. Third, the risk of a metachronous contralateral tumor may reflect unexplained differences in UTUC rates between ethnicities. For example, in the Chinese population, the incidence of UTUC accounted for up to 31% of all urothelial carcinoma^16^. This rate is much higher than published rates for the western population^17,18^.

There are currently the two main accepted hypotheses about the multifocality and recurrence of urothelial carcinoma. One is the field cancerization concept^19,20^, proposing that urothelial carcinogen exposure results in the development of an independent multiclonal tumor. The alternative hypothesis is intraluminal seeding theory^21^, in which the multifocality or recurrence of urothelial carcinomas occurs due to intraluminal implantation deriving from the monoclonal origin of tumor cells. Interestingly, in this cohort, we observed that tumor local excision might increase the risk of metachronous contralateral UTUC compared with RNU, although differences were not statistically significant in the multivariable model. The rarity of this disease limits the ability to assess for significant differences. We suggest patients with UTUC should be carefully considered for endoscopic resection. However, we cannot favor which hypothesis that has a more important effect on the development of metachronous contralateral UTUC in this cohort due to lack of more detailed clinical information of the patient, such as clinical data about vesicoureteral reflux, ureteroscopy, carcinogenic toxins exposure, renal function, multifocality, and so on. Future studies should further elucidate the potential mechanisms for developing metachronous contralateral UTUC.

Understanding the incidence of metachronous contralateral UTUC varies from person to person, and identifying the high-risk population of developing a metachronous contralateral tumor is important for appropriate surveillance and management of UTUC patients. Our findings from this study indicated that younger patients with unilateral UTUC and small tumor size had significantly increased risk of developing metachronous contralateral tumors. It is possible that this relationship exists partly because younger patients have more time to develop new contralateral tumors and thus could have a higher probability of developing a metachronous contralateral UTUC. In addition, we also found that unilateral UTUC patients with the presence of bladder cancer have a significantly greater risk of contralateral disease than those without bladder tumors. The status of bladder cancer has been considered as an important risk factor for metachronous contralateral UTUC in the previous reports^8,10,11^. Furthermore, we found 70.1% of patients with contralateral disease developed within 5 years and 10.4% were discovered ≥ 10 years after the initial diagnosis. These findings may provide an appropriate surveillance strategy for detection of the development of contralateral UTUC in most patients. Therefore, we suggest that clinicians should provide individualized cancer surveillance for high-risk UTUC patients, including younger patients, cases who had small tumor size and the history of bladder cancer. However, we should be aware that the overall risk of developing a new metachronous contralateral UTUC is relatively low, since the overall 30-year cumulative incidence in our cohort was 2.6%.

Previous studies have emphasized the good survival outcome of patients with metachronous contralateral UTUC^9,12^, but the published reports are limited by small numbers and usually derived from referral centers. Our results also support that developing metachronous contralateral UTUC did not compromise overall survival compared with that of unilateral UTUTC patients regardless of tumor stage. However, the statistically significant association between tumor stage and survival emphasizes the importance of early diagnosis of contralateral UTUC. Furthermore, our findings should be viewed within the context of most patients presented with localized disease at the time of the diagnosis of metachronous contralateral UTUC.

The major strength of the current study was its large sample size, allowing us to perform statistical analyses of a relatively larger group of metachronous contralateral UTUC. Importantly, the current analysis mainly focused on the American population, showed that the incidence and risk factors of this population might be different from patients in Eastern Asia, such as China and Korea. In addition, this population-based study can avoid selection bias that may affect patients derived from referral centers. Therefore, the associations and predictors of metachronous contralateral UTUC we describe have certain representativeness of the general population.

Our study also has some limitations, including the lack of detailed clinical information about tumor multifocality, and the lack of data on adjuvant treatment. Moreover, we have no detailed information on clinical risk factors for developing a contralateral metachronous UTUC, such as a history of renal transplantation, or renal insufficiency. Additionally, because we include only pathologically confirmed disease, these patients without pathologically diagnostic confirmation were not captured, which may result in underreporting of metachronous, contralateral UTUC. Finally, it should also be noted that due to differences in the TNM system, tumor grading methods, and patient ethnicity, this study was limited by the nature of SEER database, which might lead to different results for patients in other cohorts.

In conclusion, our findings indicate that patients with UTUC have a significantly increased risk of developing a metachronous contralateral tumor, and have a relatively modest 30-year cumulative risk (2.6%) of contralateral, metachronous UTUC. Furthermore, we found the development of contralateral UTUC was not associated with worse survival. Importantly, our results showed that the risk factors of contralateral disease included younger age, small tumor size, and bladder cancer presence. Consequently, our findings provide reasonable evidence that clinicians should encourage high-risk patients to accept intensive surveillance for UTUC screening.

## Data Availability

The datasets generated during the current study are available from the corresponding author on reasonable request.
